# Histological evaluation of duodenal biopsies from coeliac patients: the need for different grading criteria during follow-up

**DOI:** 10.1186/s12876-015-0361-8

**Published:** 2015-10-14

**Authors:** Luca Elli, Enea Zini, Carolina Tomba, Maria Teresa Bardella, Silvano Bosari, Dario Conte, Letterio Runza, Leda Roncoroni, Stefano Ferrero

**Affiliations:** 1 Center for the Prevention and Diagnosis of Coeliac Disease, Gastroenterology and Endoscopy Unit - Fondazione IRCCS Ca’ Granda Ospedale Maggiore Policlinico, Via F. Sforza 28, 20100 Milan, Italy; 2 Pathology Unit, Fondazione IRCCS Ca’ Granda Ospedale Maggiore Policlinico, Via F. Sforza 28, 20100 Milan, Italy; 3 Department of Pathophysiology and Transplantation, Università degli Studi di Milano, Via Festa del Perdono 7, 20100 Milan, Italy; 4 Department of Biomedical, Surgical and Dental Sciences, Università degli Studi di Milano, Via Festa del Perdono 7, 20100 Milan, Italy

**Keywords:** Coeliac Disease, Small Intestine, Histopathology, Malabsorption

## Abstract

**Background:**

Coeliac disease is characterised by villous atrophy, which usually normalises after gluten withdrawal. Sometimes the revaluation of duodenal histology is required during follow-up, even if the methodology for comparing duodenal histology before and after introducing a gluten-free diet is not yet established. Our aim was to evaluate a novel criterion to compare duodenal histology in coeliac disease before and after gluten withdrawal.

**Methods:**

Duodenal biopsies from coeliac patients were retrospectively reviewed to compare duodenal histology at diagnosis and after at least one year on a gluten-free diet. Two different methods were used: the first was represented by the classical Marsh-Oberhuber score, the second compared the areas covered by each Marsh-Oberhuber grade and expressed as percentages, the final grade being calculated from the analysis of ten power fields per duodenal biopsy.

**Results:**

Sixty-nine patients (17 males 52 females, age at diagnosis 36 ± 15 years) who underwent duodenal biopsies, were considered. According to the classical Marsh-Oberhuber scale, 32 patients did not present atrophy during follow-up while 37 showed duodenal atrophy, among whom 26 improved the grade of severity and 11 retained the same one. Of these latter, according to the second method, eight patients were considered improved, two showed a worsened duodenal damage and only one remained unchanged; the evaluation changed in 91 % of cases.

**Conclusions:**

The proposed semi-quantitative approach (*i.e.* the second method) for the evaluation of histology at follow-up provides additional information about the progression/regression of the mucosal damage.

**Electronic supplementary material:**

The online version of this article (doi:10.1186/s12876-015-0361-8) contains supplementary material, which is available to authorized users.

## Background

Coeliac disease (CD) is a common (1:100) autoimmune disorder of the small bowel: CD is triggered by the ingestion of gluten in genetically susceptible subjects carrying the HLA type II DQ2 and/or DQ8 haplotypes [1–3]. CD is characterised by a wide variability of symptoms, ranging from severe malabsorption to subclinical or silent pictures as observed during screening programmes [4]. CD diagnosis is based on: the detection of specific auto-antibodies (anti-transglutaminase type 2 IgA) and compatible findings at duodenal histology, including surface enterocyte damage, increased intraepithelial lymphocytes (IELs), crypt hyperplasia and villous atrophy being considered the principal hallmark [4].

Although the aforementioned mucosal alterations are not completely specific to CD, histology is considered the gold standard for its diagnosis in adults: therefore the close collaboration between clinicians and pathologists is required as well as the availability of a standardised classification in order to obtain correct comparable diagnoses [5]. In 1990 Marsh [6] proposed a classification describing four types of lesions named: type 1 or infiltrative with an increased number of IELs, type 2 or infiltrative-hyperplastic characterised by an increased number of IELs plus crypt hyperplasia, type 3 or flat/destructive with the above lesions plus villous atrophy, and lastly type 4 or atrophic/hypoplastic with a normal number of IELs and crypts and absence of villi. According to the Marsh criteria revision proposed by Oberhuber et al. [7] in 1999, the atrophic type 3 lesion was subdivided in 3a, 3b and 3c in the presence of mild, marked and total villous atrophy respectively (Marsh-Oberhuber classification). Another classification classifies CD damage simply as atrophic and non-atrophic [8].

A further factor, which strongly facilitates CD diagnosis, is the response to a gluten-free dietary regimen (GFD) usually leading to the improvement/disappearance of symptoms together with the normalisation of antibody titres and duodenal histology. Therefore, in the majority of cases, a second histology to confirm the diagnosis is not required when the clinical status is silent and auto-antibodies negative. A great amount of data has been published on the histological criteria for CD diagnosis: less information is available though about the histological modifications during GFD and the ways to compare histological reports before and after GFD. This is a crucial point for both the correct classification of CD and related patient management in view of the recent data showing the persistence of villous atrophy in spite of the complete adherence to GFD [9, 10].

Therefore, the aim of our study was to highlight the histology of the treated coeliacs, to show how it would vary within a biopsy piece and thus to apply a new proportional method to evaluate the duodenal biopsies from coeliac patients at diagnosis and during follow-up.

## Methods

### Patients and study design

The retrospective series included CD patients attending the Centre for the Prevention and Diagnosis of Coeliac Disease and the Pathology Unit of Fondazione IRCCS Ca’ Granda-Ospedale Maggiore Policlinico in Milan, Italy. The “Centre for the Prevention and Diagnosis of Coeliac Disease” is a tertiary referral centre collecting cases from the Lombardia region, northern Italy. The studied cohort was collected from 1^st^ January 2013 to the 1^st^ June 2013 included patients diagnosed between 2000 and 2012. CD was diagnosed in accordance with the internationally accepted criteria [5] and all the consecutive patients with duodenal histology at diagnosis (following a gluten-containing diet) and after at least one year on GFD were enrolled. Patients with IgA deficiency were excluded from the study. The CD onset was classified as: classical (diarrhoea, weight loss, longitudinal growth retardation), non-classical (dyspepsia, anemia, hypertransaminasemia, osteopenia, etc.), associated with the presence of dermatitis herpetiformis or during a screening programme on first-degree relatives [11].

The study was carried out in accordance with the national law on retrospective studies (laws 571/2013 and 572/2013) and approved by the “Fondazione IRCCS Ca’ Granda Ethics Committee”. In accordance with the regulatory laws 571/2013 and 572/2013 the need for patients consent was waived.

### Upper gastrointestinal endoscopy and histopathological evaluation

Upper gastrointestinal endoscopy and biopsies were performed with a flexible endoscope (Olympus, Japan) and standard bioptic forceps (Boston Scientific, USA). Four correctly oriented biopsy specimens were taken from the duodenum for every patient. Briefly, the samples were oriented on adhesive filter paper, fixed in a 10 % formalin buffer and paraffin embedded. Sections (3 μm thick) were cut from each block and hematoxylin/eosin stained. IELs were counted by means of anti-CD3 immunohistochemical staining (monoclonal mouse anti-human CD3 clone F7.2.38, Dako, Italy) [12].

The duodenal histology was independently evaluated by two expert pathologists (SF and SB), who were unaware of the patient’s disease history and clinical findings. An Olympus DMD 108 microscope was used for analysis. Power fields (PFs) 200X manignification (650x490 μm) were acquired for examinations Two different methods were used to define duodenal injury. The first method was the classical Marsh-Oberhuber scale (MO method) [7]. Briefly, the subsequent criteria were used: i) villous atrophy was classified as mild when villi were short or blunted (3a), marked when only short tent-like villi were seen (3b) and total when villi were absent (3c); ii) crypt hyperplasia was considered when dealing with more than two crypts for each villus; iii) IELs were counted on anti-CD3 stained sections and the number of 25 lymphocytes/100 enterocytes was used as cut-off. When the above mentioned alterations were absent, the duodenal mucosa was considered normal (0 type) [8]. In the second method, named “EF method” and elaborated by two of the authors (LE and SF), the whole biopsies were observed by each pathologist. When heterogeneity in morphology and/or IELs number was observed, ten PFs for each biopsy piece were considered and analysed according to the Marsh-Oberhuber scale. The different grades of lesions were recorded as percentages. For each patient the quantification of the different grades observed rather than a single one (the worst) was then obtained.

In case of discordance between the two pathologists the mean value was considered. Supplementary sections were cut to reach the appropriate number of PFs when needed. The report based on the application of the EF method, described the different proportions of the area covered by each Marsh-Oberhuber grade, while the MO method considered only the worst one.

An example of the EF analytical method is reported in the Additional files 1, 2 and 3, where two (Additional files 2 and 3) ten-PF sequences are provided.

### Statistical analysis

All the assumptions were verified by software SPSS version 18 (IBM SPSS, Italy), and a *p* value <0.05 two tails was considered as statistically significant. Kolmogorov-Smirnov’s test was used to assess the normal distribution of the data. Continuous variables were analysed with the ANOVA one-way variance test. The significance level was further verified by multiple-comparison analysis with Tukey’s test. Categorical variables were compared by *χ*^2^ (chi square) or Fisher’s exact tests. Correlations were analysed by Pearson or Spearman’s tests.

In order to evaluate the level of agreement between the two pathologists, the kappa statistic was used with values near zero indicating chance agreement only, while values near the maximum of 1 being consistent with perfect agreement: a good acceptable agreement was considered for *k* >0.80 [8]. Data were shown as mean ± standard deviation.

## Results

### Clinical and histological characteristics of the coeliac patients examined

**Fig. 1:**
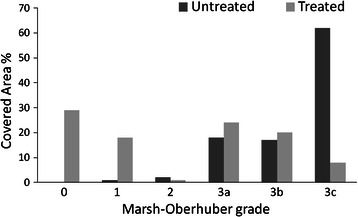
Distribution of duodenal damage (Marsh-Oberhuber grading) in untreated (following a gluten containing diet) and treated (following a gluten free diet) coeliac patients applying the EF method (see Methods section)

**Fig. 2:**
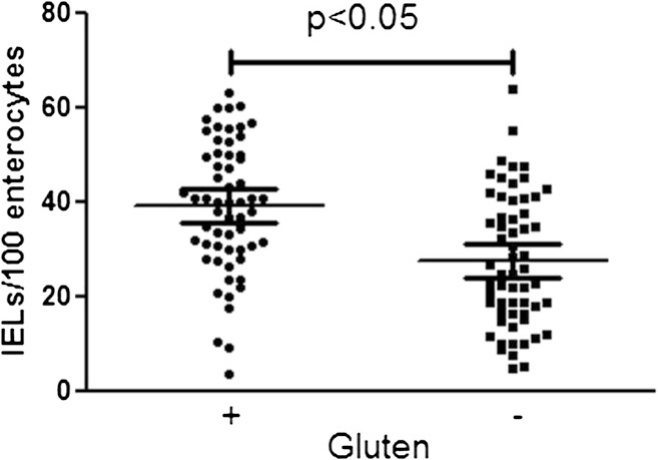
Number of duodenal intraepithelial lymphocytes (IELs) in untreated (gluten +) and treated (gluten -) coeliac patients. Means and standard deviations are reported in the plot

The clinical and histological characteristics of 69 consecutive CD patients satisfying the entry criteria were retrospectively analysed and a total 2,760 PFs observed. The study cohort was composed by 17 (24 %) males and 52 (76 %) females, with a mean age at diagnosis of 39 ± 15 years (range 14–69), following a gluten-free regimen for a 4 ± 3 years mean period (range 1–13); 12 (17 %) patients presented an associated autoimmune disease (Hashimoto’s thyroiditis in 9 cases, alopecia in two and Sjogren’s disease in one). As to clinical presentation 34 (50 %) patients had a classical onset, 25 (36 %) were non-classical, 7 (10 %) had been diagnosed during familial screening and 3 (4 %) presented with dermatitis herpetiformis. According to the MO method, Table 1 details the histological grade at diagnosis and during GFD. Of interest, the worst histological findings were observed in patients with non-classical clinical onsets including familial screening and dermatitis herpetiformis. In detail, 3c grades more often occurred in the non-classical group than in the classical one (96 % *vs.* 74 %, *p* <0.05). Gender and age at diagnosis did not influence the MO score. As from Table 1, at diagnosis 72 % CD patients were graded 3c; GFD achieved a considerable redistribution of MO scores towards mild atrophy (3a) or non-atrophic lesions (0, 1 and 2); 32 CD patients resulted non-atrophic, mild (3a) and moderate (3b) atrophy increased from 20 to 29 cases and severe atrophy (3c) decreased from 49 to 8. Similarly, percentage of atrophy decreased after gluten withdrawal applying the EF method (Fig. 1). Of relevance, 37 (53 %) patients retained an atrophic picture when analysed according to the MO method (Table 1). As shown in Fig. 2, IELs significantly decreased during GFD. No demographic factors among those examined (type of presentation, gender and age) significantly correlated with the improvement of the duodenal mucosa; of interest, the GFD duration failed to influence the histological findings (see next paragraph).

**Table 1:** Histological findings of 69 coeliac patients at diagnosis and during their gluten-free diet (GFD), according to the Marsh-Oberhuber classification

Marsh-Oberhuber grade	At Diagnosis		During GFD	
	*N*	(%)	*n*	(%)
0	0	(0)	20	(29)
1	0	(0)	11	(17)
2	0	(0)	1	(1)
3a	10	(14)	17	(25)
3b	10	(14)	12	(17)
3c	49	(72)	8	(11)

*GFD* gluten-free diet

### Analysis of coeliac patients with persistence of duodenal atrophy

**Fig. 3:**
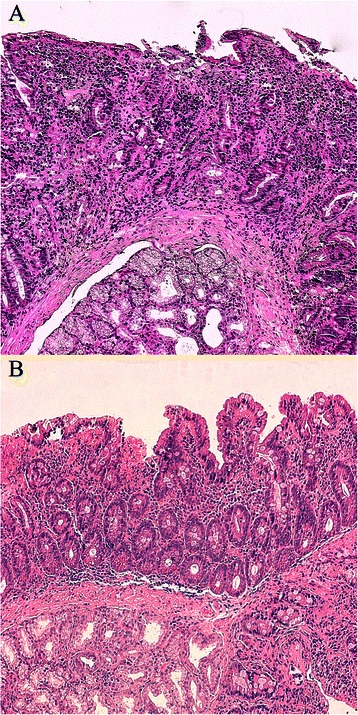
Duodenal histology from a coeliac patient on a gluten-containing diet showing a homogenous Marsh-Oberhuber 3c lesion (**a**). In **b**, the duodenal histology from the same patient following a gluten-free diet is reported showing different grades of damage (3c on the left side of the panel and 3a on the right side). Using the Marsh-Oberhuber classification the two biopsies will be classified as 3c although presenting a different pattern

According to the MO method, 37 treated CD patients (9 males and 28 females, age at diagnosis 39 ± 17 years, GFD duration 3 ± 3 years) retained duodenal atrophy. Again, the demographic parameters of this group did not show any statistical difference compared to the 32 treated CD patients (8 males and 24 females, age at diagnosis 33 ± 12 years, GFD duration 4 ± 3 years) without duodenal atrophy. Out of the 37 CD patients with duodenal atrophy, the sub-grade improved from severe to moderate/mild atrophy or from moderate to mild in 26 patients, whereas in the remaining 11 patients (11 females, age at diagnosis 40 ± 18 years, GFD duration 4 ± 4 years) the level of atrophy did not change. Again, no demographic or clinical differences were found between these two groups. On analysing the 11 unchanged patients according to the EF method, their histological pattern appeared more complex than that described by the traditional MO method (the relevant results are detailed in Table 2 and Fig. 3). As from Table 2, the second analysis provided different histological results; in detail, 8 treated CD patients were considered improved, 2 worsened and only one unchanged.

**Table 2:** Histological analysis results obtained with the EF method (see Methods section) in 11 coeliac patients presenting the same grade before and after gluten withdrawal when analyzed by the classical Marsh-Oberhuber scoring system

	Marsh-Oberhuber grade (%)^a^												
	At Diagnosis				During GFD								
Patient	3a	3b	3c	IELs %	0	1	2	3a	3b	3c	IELs %	Years after diagnosis (*n*)	Outcome
1	0	0	100	31	0	0	0	15	0	85	48	3	I
2	0	0	100	23	0	0	0	50	35	15	45	2	I
3	10	90	0	47	0	0	0	0	95	5	64	13	W
4	0	0	100	49	50	20	0	0	0	30	31	9	I
5	100	0	0	4	20	0	5	75	0	0	12	1	I
6	0	100	0	53	0	0	0	0	100	0	35	7	U
7	0	40	60	27	0	0	0	0	0	100	34	1	W
8	0	0	100	56	0	0	5	0	20	75	61	1	I
9	0	0	100	45	0	0	0	0	40	60	38	6	I
10	0	0	100	55	0	10	0	0	30	60	32	1	I
11	70	30	0	42	0	0	0	20	80	0	46	2	I

^a^ % of area covered, *IELs* intraepithelial lymphocytes, *GFD* gluten-free diet, *I* improved, *MO* Marsh-Oberhuber, *W* worsened, *U* unchanged

The k test for the inter-observer agreement resulted 0.91 ± 0.25.

## Discussion

The present study focused on the evaluation of duodenal biopsies from CD patients before and after GFD. The application of a novel approach (EF method) instead of the classical qualitative one (MO method) led to significant changes in the histological reports, enabling the distinction of patients with different evolution outcomes of the duodenal damage.

Although different systems have been proposed to classify the CD duodenal damage (*i.e.* the Marsh score [13] and its modification by Oberhuber [7] and the recently introduced Corazza-Villanacci grading [8]), no approach to follow a CD patient’s evolution has until now been proposed and the dietary state has been rarely considered. The introduction of a new method appeared necessary in case of unchanged responses under the Marsh-Oberhuber classification (the worldwide referral). Previous studies analysed the number of IELs and the villus height/crypt depth ratio [14–16] or the villous area [17] and their relationship to gluten intake; however, such parameters appear difficult to apply in a clinical setting, directly correlated with the Marsh grade, and thus do not prove useful when this grade is unmodified.

At present, the evaluation of duodenal histology does not differ whether CD patients are or are not on a GFD regimen, as the analysis is mainly focused on spotting the presence of an architectural damage (atrophy). These parameters have been summarised in the Marsh-Oberhuber classification and strongly influence the specialists in their clinical/therapeutic decisions, especially when the histological pattern is unmodified [18]. However, the effects of GFD can be interpreted for further diagnostic clues. Our findings suggest that in adult CD patients, even those on a strict dietary regimen, the histological response can be slow (or absent) and that mucosal atrophy does not always turn into complete/partial normalisation [9, 10]. This result has been reported by different studies [19] and corroborated by data from videocapsule endoscopy studies, consistent with the persistence of macroscopic signs of intestinal atrophy with a skip fashion [20]. Again, during a gluten-free regimen, serological tests (*i.e*. anti tissue transglutaminase IgA) are associated with clinical responses but do not correlate with the histological improvement of the duodenal mucosa (mucosal healing) [21]. The gap between serological and endoscopic/histological findings represents a potential problem for patient management in case of persistent mucosal atrophy. In their study Kaukinen et al. [21] underlined that the occasional ingestion of gluten is not associated with any increase in anti-transglutaminase IgA titers. Interestingly, another series reported only minimal mucosal changes after the controlled ingestion of gluten [22]. Moreover, there is evidence that different types of food commonly believed gluten-free, in fact contain gluten traces [23] and that an accurate diet analysis often reveals the incomplete adherence to GFD thus explaining the persistent mucosal atrophy or removing the suspicion of a refractory sprue [24, 25]. Autoptic studies [26] showed a progressive decrease of mucosal injury from the proximal to the distal tract of the small intestine; thus, the clinical picture can reflect the longitudinal involvement of the small bowel rather than the type of mucosal damage [27]. Additionally, the histological damage in naïve patients is often patchy [28, 29]. For this reason, in order to minimise the risk of under-estimation, the evaluation of at least 4 oriented duodenal endoscopic biopsies is strongly recommended.

The reason underpinning the persistence of villous atrophy in treated CD is not easy to understand, although a miRNA signature might be involved [20]. In our tertiary referral Centre, a significant proportion of CD patients were referred to undergo complete endoscopic investigations, highlighting how much the need to compare duodenal histology before and after GFD is particularly felt, in order to proceed with a watchful follow-up in selected cases. The above data led to a relevant question, i.e. does the incomplete normalisation of the intestinal mucosa represent a risk factor for CD complications? If yes, the current data and international guidelines on CD follow-up may suggest the need for further biopsies after diagnosis, at least until some mucosal healing is present. Therefore, the opportunity of quantifying the damage and evaluating its trend would be important.

Since some symptoms or complications of CD are correlated with mucosal damage (*e.g.*, osteoporosis) [30], and other ones are caused by autoantibodies (*e.g.*, infertility) [31], mucosal biopsy and serological tests should be considered complementary, and the quantitative evaluation of mucosal atrophy proves helpful in the evaluation of clinical findings and patient management.

When applying the EF method to patients with persistent duodenal atrophy, 91 % of cases changed the histological assessment, with a regression in 72 % or even a progression in 18 %.

Whilst the simple detection of atrophy in the duodenal mucosa of naïve patients is enough to make a correct diagnosis (as in the Corazza-Villanacci grading), the context dramatically changes when dealing with patients on GFD, for whom the main question is about the presence or not of any histological improvement. The answer to this question requires a more specific scoring system. A possible problem is represented by the loss of inter-observer agreement when the number of grades increases. In our study the k test showed good agreement levels but we suggest that this type of analysis should be used in tertiary settings by expert pathologists.

Moreover, the possible availability of drug-based therapies for CD in the next future would add to the interest in systems, such as the EF method, aimed at comparing duodenal histology before and after GFD, In such a case, the judgment about the improvement of duodenal atrophy will be pivotal in the therapeutic decision-making process [2].

However, some weak points of the study need to be discussed. The study has a retrospective design and thus prospective series are necessary to evaluate the long term clinical impact and prognosis of patients with an unchanged MO classification but showing a different finding with the EF one; in particular, if unchanged or worsened duodenal histology with the EF method has an increased risk of complications. Interpretation of duodenal histology could be challenging due to the patchy nature of CD damage in some circumstances and the need of a strict collaboration between clinical gastroenterologist and pathologist is essential. Moreover, the EF comparison between duodenal histology at diagnosis and during follow up could be performed only in case of correct orientation of the samples (not always possible). In our study the time between diagnostic and follow-up biopsy is not uniform and a dietetic questionnaire was impossible to ascertain the correctness of the GFD.

Noteworthy, this retrospective study was conducted in a tertiary referral centre where complicated or challenging cases are managed; thus, a different result could be present in other scenarios where dedicated and/or expert personnel are absent. Another point about the EF method is the cost analysis in the light of a possible clinical benefit; in fact, the EF method is time consuming and imperfectly oriented biopsies could introduce the necessity of an increased number of samples per patient.

## Conclusions

Overall, the present results indicate that duodenal biopsies should be analysed with different methods depending on the clinical setting: is the naive patient affected by CD *i.e.* does the patient present any duodenal atrophy? In this case the pathologist can apply a simple score that indicates whether the subject presents any atrophy (Marsh 3 grade) or mild enteropathy (Marsh 1 or 2 grades) or has a normal duodenum (Marsh 0). When dealing with treated CD patients the clinical question changes to: has histology improved or not? In other words, has the duodenal mucosa shown the “tendency” to improve/normalise? In such a setting, a careful approach comparing the biopsies acquired during gluten ingestion with those taken on GFD, as with the proposed EF method, would precisely indicate the patient’s evolution.

## Additional file

Additional file 1: **Description of the EF method.** (DOC 25 kb) Additional file 2: **PFs from a duodenal biopsy of a coeliac patient on a gluten-containing diet.** (JPEG 2249 kb) Additional file 3: **PFs from a duodenal biopsy of the same coeliac patient presented in Additional file**2**, on a gluten-free diet.** (JPEG 1762 kb)

## Abbreviation

CD: Coeliac Disease
EF: Elli-Ferrero
GFD: Gluten Free Diet
IEL: Intraepithelial Lymphocytes
MO: Marsh-Oberhuber
PF: Power Fields
