# Supplementary material for: Histological evaluation of duodenal biopsies from coeliac patients: the need for different grading criteria during follow-up
Source: BMC Gastroenterol. 2015 Oct 14;15:133. doi: 10.1186/s12876-015-0361-8 (PMC4604755; doi:10.1186/s12876-015-0361-8)
Supplement: Additional file 1: — Description of the EF method. (DOC 25 kb) [file 12876_2015_361_MOESM1_ESM.doc]

**Supplementary Information**

Additional files 1 and 2 show 20 randomly selected power fields (PFs, magnification 200x) from two different duodenal biopsies from the same coeliac patients, before and on gluten-free dieting.

Additional file 1. PFs from a duodenal biopsy of a coeliac patient on a gluten-containing diet. Following the classical Marsh-Oberhuber score (named MO method in the manuscript) the histological grade was classified as 3c; with the proposed method (named EF in the manuscript), the same sample is described as composed of 55% 3c and 45% 3b.

In detail, field “a” was described as 100% 3c, field “b” 50% 3b and 50% 3c, field “c” 100% 3b, field “d” 50% 3b and 50% 3c, field “e” 100% 3c, field “f” 100% 3c, field “g” 50% 3b and 50% 3c, field “h” 100% 3b, field “i” 100% 3b and, lastly, field “l” 100% 3c

Additional file 2. PFs from a duodenal biopsy of the same coeliac patient presented in Figure 1, on a gluten-free diet. Applying the Marsh-Oberhuber score (MO method) the biopsy would be classified 3c as well; differently, with the EF method, the damage is described as composed of 10% 3c, 20% 3b and 70% 0 demonstrating an important informational improvement.

In detail, field “a” was described as 100% 0, field “b” 100% 3b, field “c” 100% 0, field “d” 100% 3c, “e” 100% 0, field “f” 100% 0, field “g” 100% 0, field “h” 100% 0, field “i” 100% 3b and field “l” 100% 0.

The mean of the percentages from other three biopsies per patients was given in the main report.
