# Supplementary figures and images for: Histological evaluation of duodenal biopsies from coeliac patients: the need for different grading criteria during follow-up
Source: BMC Gastroenterol. 2015 Oct 14;15:133. doi: 10.1186/s12876-015-0361-8 (PMC4604755; doi:10.1186/s12876-015-0361-8)

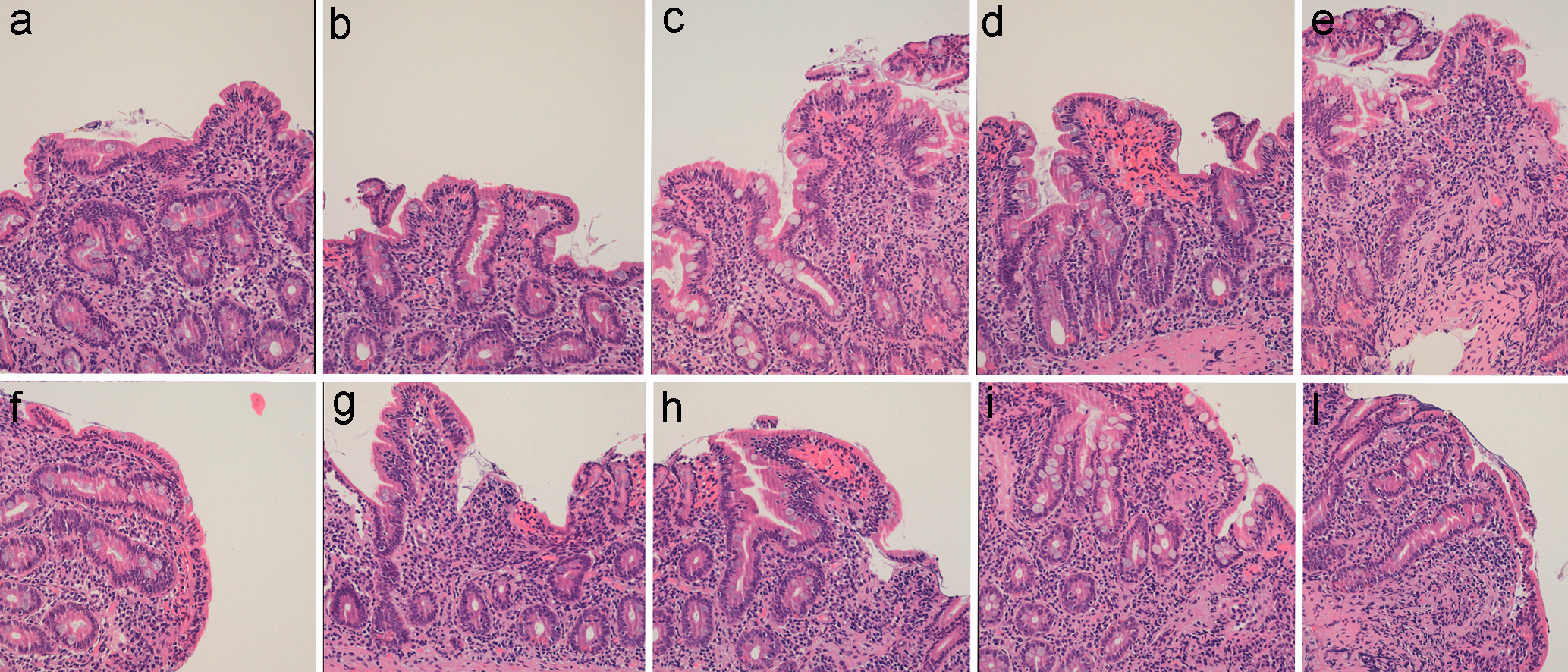

Supplement: Additional file 2: — PFs from a duodenal biopsy of a coeliac patient on a gluten-containing diet. (JPEG 2249 kb) [file 12876_2015_361_MOESM2_ESM.jpeg]

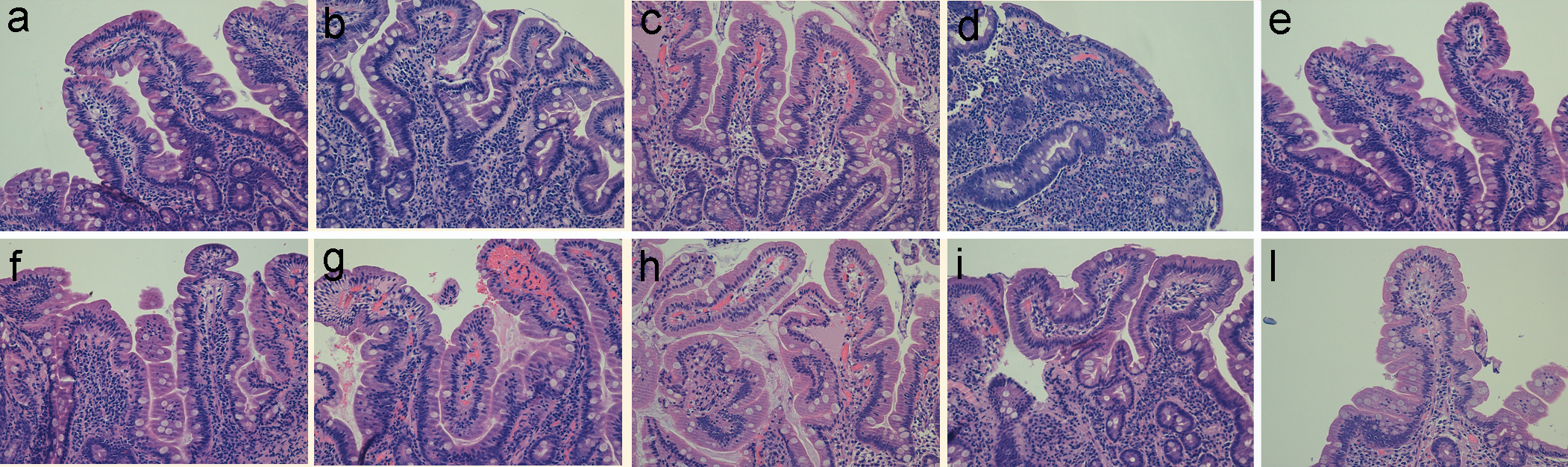

Supplement: Additional file 3: — PFs from a duodenal biopsy of the same coeliac patient presented in Additional file2, on a gluten-free diet. (JPEG 1762 kb) [file 12876_2015_361_MOESM3_ESM.jpeg]
